# Influence of two different feeding strategies in the dry period on dry matter intake and plasma protein peroxidative and antioxidative profile during dry period and early lactation

**DOI:** 10.1186/s12917-020-02347-x

**Published:** 2020-05-13

**Authors:** Yasmin Gundelach, Beate Streuff, Monika Franczyk, Marta Kankofer, Martina Hoedemaker

**Affiliations:** 1 Clinic for Cattle, University of Veterinary Medicine Hannover, Foundation, Hannover, Germany; 2 Present Address: Educational and Research Centre for Agriculture, Haus Düsse, Ostinghausen, 59505 Bad Sassendorf, Germany; 3 Department of Biochemistry, Faculty of Veterinary Medicine, University of Life Sciences, Lublin, Poland

**Keywords:** Dairy cow, Dry period, Oxidative stress, Diet, Dry matter intake

## Abstract

**Background:**

Dairy cows undergo dramatic changes in endocrine and metabolic status around parturition and in early lactation. Meeting the nutritional requirements of transition dairy cows is important for animal health, production and animal wellbeing. Dry cow feeding and managing play an essential role in this. The changes in metabolism of periparturient cows also lead to a rise in the production of oxidising agents, leading to oxidative stress. The relationship between dry cow diet composition and oxidative stress has received little research attention so far. In the present study, the influence of two different dry cow feedings (single diet with medium energy content over the whole dry period versus traditional two-phase diet with a low-energy “far-off” ration and a high energy “close-up” ration) on dry matter intake, energy intake and plasma protein peroxidative and antioxidative profile was investigated.

**Results:**

The examined parameters revealed a dynamic profile within the experimental period. Dry matter intake (DMI) did not differ between groups. However, there was a time and a group x time interaction effect: Group 1 (“one-phase”) had a very constant DMI with a slow and even decrease until calving. In Group 2 (“two-phase”), an initial increase in DMI two weeks antepartum (a.p.) was followed by a sharp drop at week 1 a.p.. The highest total antioxidant capacity and sulfhydryl residue concentration was noted at partus. In contrast, concentration of formylokinurenine and bityrosine bridges as representatives of protein peroxidation were lowest at parturition. The time course of formylokinurenine and bityrosine bridges showed parallels to the DMI. The contents of sulfhydryl groups, formylokinurenine and total antixoxidant capacity did not differ between groups. In contrast, concentration of bityrosine bridges was always higher in Group 2 compared with Group 1 and these differences were statistically significant at week 3 a.p., week 2 a.p., week 1 a.p. and at parturition.

**Conclusion:**

The results of our study suggest time-related changes of pro- and antioxidative plasma parameters. Different dry cow feeding affected antepartal DMI. Furthermore, DMI and diet compositions seemed to have an influence on plasma protein peroxidative profile and activity of antioxidative defence.

## Background

The dry period, in particular the transition period, is characterised by dramatic changes in endocrine and metabolic status. The increase in energy demands required after calving for milk production in high-yielding dairy cows and the concomitant insufficient dry matter intake (DMI) lead to a negative energy balance in early lactation [1, 2]. Dry cow feeding and managing should prepare the cow for the next lactation as well as possible. Nutritional strategies have varied during the last decades. Traditionally, the dry period is divided into two feeding periods. In the “far-off” period, cows are fed a high-fibre, low-energy diet for the first weeks of the dry period. This is followed by a “close-up” period, typically 3 weeks long, during which cows are fed a diet with higher energy densitiy. As dry matter intake drops markedly before calving, increase in energy content in the close-up period may improve production and health in early lactation [3, 4]. In addition, it was shown that DMI was increased in the prepartum period when higher energy diets were fed [3, 5, 6]. In contrast, several authors have reported that overfeeding especially during the early dry period might lead to appetite depression and an increased incidence of health disorders [7–10]. Therefore, feeding cows a single controlled energy diet prepartum had beneficial effects on metabolic health [8, 10, 11]. Dry matter intake remained more constant in the transition period, when high-straw, low energy diets were fed [7]. In addition, single-group systems would have the advantage of eliminating a group change, which may then decrease social stress [12, 13]. Due to the high metabolic demands and dysfunctional inflammatory responses, cows routinely experience substantial oxidative stress in early lactation [14, 15]. Several studies suggested that oxidative stress increased the susceptibility of dairy cows to diseases [15–17]. Sordillo and Aitken [15] hypothesised that oxidative stress during the transition period may be a major underlying cause of inflammatory and immune dysfunction in dairy cattle. Measuring the concentration of peroxidation end products is a widely used method for the assessment of oxidative stress. Proteins are extremely sensitive to the action of free radicals. The oxidative modification of proteins is faster and linear (with regards to time and concentration) than the lipid peroxidation process. Therefore, concentration of protein peroxidation products is a sensitive indicator of the action of reactive oxygen species (ROS) on the cellular components [18]. Indicators of protein peroxidation are sulfhydryl residue groups, N′-formylkinurenine and bityrosine.

A number of antioxidant-related micronutrients were shown to affect various aspects of the immune system. For example, several studies revealed significant reductions in the incidence of retained foetal membranes, cystic ovarian disease and mastitis following supplementation with vitamin E or Se [19–23]. A study by Mantovani et al. [24] investigated the modifications of blood oxidative stress indicators in response to the omission of the dry period. Their results supported the hypothesis that diet composition rather than milk synthesis and secretion effected levels of oxidative stress in the continuously milked group. The aim of the current study was to evaluate the influence of two different dry cow feedings (single diet with medium energy content over the whole dry period versus traditionally two-phase diet with low-energy “far-off” ration and high energy “close-up” ration) on dry matter intake, plasma protein peroxidative profile and activity of antioxidative defence.

## Results

**Fig. 1:**
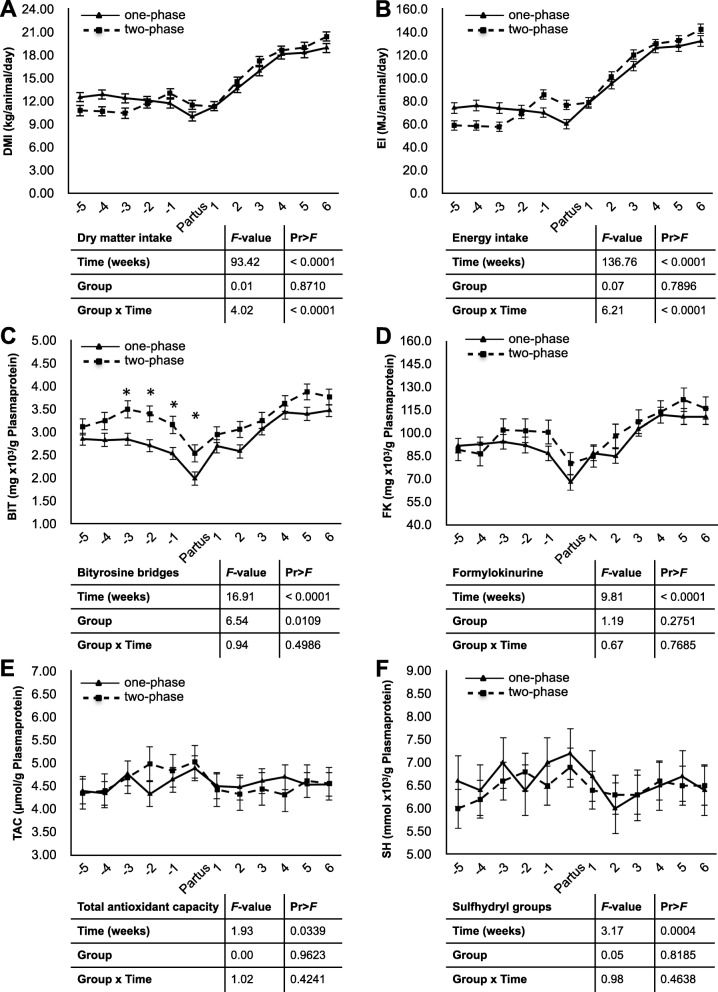
(**a**) dry matter intake (DMI) (kg/animal/day); (**b**) energy intake (EI) (MJ/animal/day); Mean concentrations ± SE of [**c**] bityrosine bridges (BIT) (mgx10^3^/g Plasmaprotein); (**d**) formylkinurine (FK) (mgx10^3^/g Plasmaprotein); (**e**) total antioxidant capacity (TAC) (μmol/g Plasmaprotein); (**f**) sulfhydryl residues (SH) (mmolx10^3^/g Plasmaprotein) in blood plasma of “one-phase” and “two-phase” fed cows from five weeks a.p. up to six weeks p.p.. Results of the SAS mixed model indicating significant (p<0.05) differences regarding the fixed effects of group, time and group × time are shown in the respective tables under the graphs. * indicates significant differences between groups

The least squares means of antioxidant and oxidant blood concentrations as well as dry matter intake and energy intake are depicted in Fig. 1. It illustrates the time pattern of DMI, energy intake, bityrosine bridges (BIT), formylokinurenine (FK), total antioxidant capacity (TAC) and sulfhydryl residue (SH) (*P*-values of temporal patterns are mentioned in Additional file 1).

### Dry matter- and energy intake

Dry matter intake did not differ between groups, but a time and a group x time interaction effect were evident (Fig. 1 a). In Group 1, DMI decreased slowly during the dry period and reached the lowest value at partus. Comparing week 5 a.p. with the following antepartum values, this decrease was significant at the time of partus (*P* < 0.05). Postpartum (p.p.) DMI increased again. The time course of DMI in Group 2 revealed a slight decrease up until week 3 a.p. followed by a significant increase 1 week a.p. coinciding with feeding the more energetic ration. As in Group 1, DMI decreased in the last week before parturition and started to increase from week 2 p.p. onwards (*P* < 0.05).

Energy intake showed the same time and interaction effect in both groups as DMI (Fig. 1 b). However, in Group 2, antepartum energy intake increased significantly already 2 weeks a.p.. Postpartum energy intake increased in group 1 at week 1 p.p. compared with parturition, whereas in Group 2, it did not increase until week 2 p.p. (*P* < 0.05).

### Bityrosine bridges

The concentration of bityrosine bridges showed a clear time and group effect, but no group x time interaction (Fig. 1 c). In Group 1, BIT decreased slowly until parturition (week 5 a.p. vs. parturition: *P* < 0.05). In Group 2, BIT increased from week 5 a.p. to week 3 a.p., followed by a decrease until parturition (week 5 vs. partus: *P* < 0.05). With 0.0020 mg/g plasmaprotein and 0.0025 mg/g plasmaprotein, in both groups, the lowest concentrations of BIT were detected at parturition. After parturition, BIT values increased significantly in both groups (partus vs. the following weeks: *P* < 0.05). Concentrations of BIT were always higher in Group 2 compared with Group 1. The differences were statistically significant at week 3 a.p., week 2 a.p., week 1 a.p. and parturition (*P* < 0.05).

### Formylokinurenine

For the concentrations of formylokinurenine, the time course was quite similar to the temporal pattern of BIT. No group x time interaction or group effect was observed. (Fig. 1 d). Similar to BIT, the lowest values for FK were measured at parturition (Group 1: 0.07mg/g plasmaprotein; Group 2: 0.08 mg/g plasmaprotein). Whereas in Group 1, FK concentrations at partus were significantly lower than at week 5 a.p. (*P* < 0.05), FK concentrations in Group 2 significantly increased from weeks 5 to 3 a.p. and 2 a.p. (*P* < 0.05), followed by a decrease until partus. As opposed to Group 1 and to BIT, the difference between week 5 and the day of partus was not significant (*P* > 0.05). Postpartum, the concentration of FK increased starting at week 1 p.p. in Group 1 and at week 3 p.p. in Group 2, respectively (*P* < 0.05).

### Total antioxidant capacity

For the TAC concentration means, no statistically significant effects existed between groups, nor was there a significant group x time interaction (Fig. 1 e). With respect to the temporal pattern, in Group 1, TAC concentrations did not differ antepartum and postpartum (*P* > 0.05). Cows in Group 2 exhibited a significant increase in TAC concentrations from week 5 ap to weeks − 2, − 1 and partus (*P* < 0.05).

Postpartum, the TAC concentrations at weeks 1, 2, 3 and 4 were lower than the values at partus (*P* < 0.05). Thereafter, the TAC concentrations increased again. However, they did not reach the concentrations at partus. For both groups, the highest TAC values were found at parturition.

### Sulfhydryl residue

Similar to the TAC values, there were no statistically significant differences between groups or an interaction effect, but a time effect was observed (Fig. 1 f). The highest mean concentrations in thiol groups were measured at parturition (*P* < 0.05) in both groups. The lowest SH concentrations were measured at weeks 2 p.p. and 3 p.p. in both groups.

Within Group 1, the SH concentration remained constant antepartum, but increased towards parturition (*P* < 0.05). Postpartum, SH values decreased and remained from week 2 p.p. at a significantly lower level compared with parturition (*P* < 0.05). Cows in Group 2 exhibited an increase in SH concentrations from week 5 to week 3 and the following antepartal weeks and partus (*P* < 0.05). SH concentrations were lower at week 2 p.p. and 3 p.p. compared with partus (*P* < 0.05). Even though there was a slight increase thereafter, the concentration levels remained below those at parturition (*P* > 0.05).

### Energy–corrected milk yield (ECM)

**Fig. 2:**
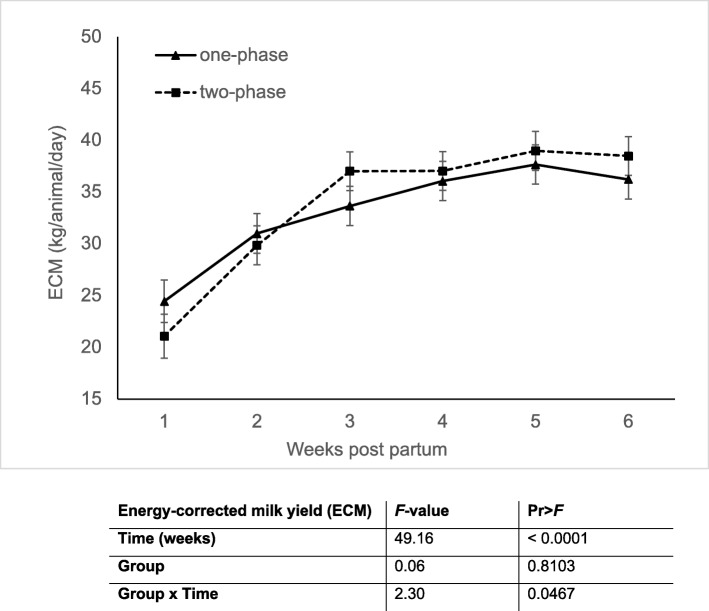
Energy-corrected milk yield (ECM, kg/animal/day) of “one-phase” and “two-phase” fed cows in the first six weeks of lactation. Results of the SAS mixed model indicating significant (*P* < 0.05) differences regarding the fixed effects of group, time and group × time are shown in the respective table under the graph

ECM did not differ between groups, but a time and a group x time interaction effect were evident (Fig. 2). In both groups, ECM increased significantly in the first 6 weeks p.p.. Cows from Group 1 have a slightly higher milk yield in the first 2 weeks, but due to the steep rise in ECM in group two, this relationship is reversed from week 3 p.p. onwards.

## Discussion

To our knowledge, this is the first reported investigation of two different diets in the dry period on plasma antioxidative (TAC, content of SH groups) and oxidative (content of bityrosine bridges and formylokinurenine) profiles.

In our study, animals in Group 1 had a very constant DMI with a slow and even decrease until calving. Several studies confirmed that a higher concentration of neutral detergent fibre (NDF) in the last weeks before calving successfully prevented a rapid decrease in prepartum DMI [7, 9]. In Group 2, an initial increase in DMI caused by feeding the high-energy close-up ratio 2 weeks a.p. was followed by a sharp drop in the last week before parturition. This finding corresponds to the observations made in other studies that increasing the nutrient density of the diet could increase DMI [6, 25]. In contrast, Vandehaar et al. [26] compared different energy and protein contents for the close-up ration and found no difference in the feed intake ante partum. Rabelo et al. [6] supposed that the greater the energy intake prepartum, the greater the magnitude of drop in DMI at parturition. In our study, the different dry cow diets did not have any significant effect on DMI and ECM postpartum. Previous studies came to very different results. For example, Doepel et al. [27] found positive effects on postpartal DMI but not improved lactation performance when feeding a close-up diet with higher energy density. This contrasts with the findings in several studies supporting a negative influence of antepartal energy overconsumption on postpartal DMI [8, 9]. In the study by Huang et al. [28] cows fed a low energy density diet during the close-up period had a significantly lower average DMI (3 weeks a.p. to 1 week a.p.) compared to cows fed a close-up diet with high density. In the last week before calving, however, the DMI for both treatments was not different. After parturition, the “low density” group had a higher average DMI (week 1 to week 5 p.p.) and milk yield (week 1 to week 10 p.p.) compared with the “high-density” group.

Protein peroxidation in plasma is an essential parameter of oxidative stress, and it can be used as an adequate marker to evaluate the level of oxidative stress in the organism. Peroxidative damage to proteins leads to the loss of their biological activity, molecule aggregation or fragmentation and finally may result in the modification of amino acid residues. Tryptophan is oxidised to formylokinurenine. As the result of the recombination of tyrosine radicals, bityrosine bridges are created [29].

Only a few existing publications focus on changes in bityrosine and formylokinurenine concentrations in plasma in the peripartal period of cattle.

In our study, the lowest concentrations of BIT and formylokinurenine were found at partus. In the study by Hanschke et al. [30], FK and BIT decreased towards parturition and increased during the postpartum period. Kankofer et al. [31] found the lowest mean concentration of BIT 1 week p.p., with a slight increase at 2 weeks and 1 week before partus. In our study, both groups had a nearly identical time course postpartum. However, antepartum, we found significant differences between groups and the time course of these parameters also showed parallels to the DMI. The slow and constant decrease antepartum of the DMI in Group 1 seemed to be associated with an almost identical course of the parameters FK and BIT. However, this relationship could not be fully confirmed for Group 2. A possible explanation for the significantly higher BIT values in Group 2 could be the change from the far-off diet to the close-up diet. More pronounced changes in DMI and the correspondingly higher fluctuations in energy and protein intake could have led to higher concentrations of BIT and FK. The higher availability of peroxidation substrates may result in a higher rate of peroxidation products. Postpartum, as previously mentioned, DMI, BIT and FK concentrations were nearly identical in both groups. The observations by Mantovani et al. [24] indicated that diet composition rather than milk synthesis may influence the concentration of oxidative stress parameters. The study by Mantovani et al. [24] investigated the modifications of blood oxidative stress indicators in response to the omission of the dry period. They found greater blood glutathione peroxidase [GPx] activities in the experimental group fed the lactation diet aimed to support milk secretion, while the normally dried off group received a two-phase dry cow diet (lower energy density and higher NDF%) formulated in order to supply the nutritional requirements by NRC [42]. GPx activities also were not different between groups after calving when lactation started, and the two groups received a similar diet. Data from Gabai et al. [32] support the hypothesis that the diet starch content may contribute to alter the control of oxidative stress. They suppose that the increase of aminoacid utilization for gluconeogenesis due to lower insulin concentration could explain the lower total plasma glutathione concentration in cows feeding a low starch diet. For lactating sheep data by Sgorlon et al. [33] indicate that rapid modification of diet composition affects metabolic and oxidative homeostasis.

In our study, the lowest values of BIT and FK in both groups were found at parturition, whereas at the same time, TAC values were the highest.

In order to examine the intensity of pro- and antioxidative status, it is useful to determine the total capacity of antioxidative agents. TAC is an integrated parameter describing the dynamic balance between oxidising agents and antioxidants within plasma [34]. In order to reveal fundamental information on the antioxidant status of animals, it is useful to define the ferric reducing ability of plasma [35].

In our study, cows exhibited a slight upward trend 5 weeks a.p. to parturition, when the concentration reached the highest value. In contrast to Group 1, for Group 2, this increase was significant and coincided with the decreased BIT concentrations, which possibly represented a compensatory reaction. Kankofer et al. [31] reported slightly different time patterns. They observed a significant increase in the TAC concentration from 4 weeks a.p. until 5 days a.p., with a sharp drop at calving and re-growth until 3 weeks p.p., before another decrease occurred.

The concentration of thiol groups can also be used to determine the level of protein peroxidation [29]. By the oxidation of SH groups, which are elements of plasma proteins including antioxidant compounds, disulfide bridges are formed, reflecting the loss of antioxidant compensatory mechanisms [36]. Previously, it has been suggested that any decrease in SH groups may be the result of proteins damage, while the increase is an expression of increased antioxidative defence [29]. Here, examined animals had a slight increase in the concentration of SH groups at parturition followed by a decrease during the postpartum period. Hanschke et al. [30] also reported a significant increase between 3 weeks antepartum and partus and a decrease again between partus and 3 weeks postpartum. Depending on the behaviour of this parameter, it can be defined as representing pro- or antioxidative status. Therefore, in our case, the slight increase until parturition represented an increased antioxidative defence which was also reflected by higher TAC values. On the other hand, the postpartal decrease in SH might be an expression of increased oxidative stress also reflected by higher BIT and FK values.

From the development of the parameters over time, it can be seen that the cows in both groups were able to manage the oxidative stress level around parturition. The slightly higher protein peroxidase products in Group 2 seemed to be compensated by an increasing total antioxidative capacity antepartum. Postpartum, when the two groups of cows were kept under the same condition and feeding regime, no group differences in protein peroxidation were observed anymore.

## Conclusion

The results of our study suggest that different dry cow diets influenced antepartal DMI and that the composition of the diet also seemed to have an impact on plasma protein peroxidative profile and activity of antioxidative defence. Due to the limited number of individuals, these results should be viewed with caution. Further studies are necessary to establish physiological ranges of antioxidative/ oxidative profiles in cows and their interrelation to DMI and different ration compositions.

## Methods

### Animals

All animal procedures were carried out in accordance with ethical guidelines for the use of animal samples, as approved by LANUV NRW (State Office for Nature, Environment and Consumer Protection; No. 84–02.05.20.12–179).

Primi- and pluriparous cows of the German Holstein breed from the Agricultural Research and Teaching Centre Haus Riswick of the Chamber of Agriculture in Northrhine-Westfalia, Germany, were used in the present study from the beginning of the dry period or, in the case of heifers, 6 weeks before the calculated calving date until 50 days postpartum. At the end of the study, the animals were returned to the normal dairy herd. The trial lasted from April 2012 until December 2012. The two experimental groups consisted of 50 animals each. The animals in each group were matched according to the number of lactation, presumed dry period length, past estimated calving interval, past 305-day milk yield, body weight and first calving date (heifers). The animals in each pair were then randomly assigned to the experimental groups. In the further evaluation for this study, 20 animals per group were randomly selected.

### Housing

Dry cows and heifers were kept in a free stall barn with raised cubicles with mattresses covered with milled straw. The two feeding groups were housed in separate compartments until calving. Approximately two days before the expected calving date, the animals were moved to straw yards. Immediately after calving, the cows were moved to different straw yards, where the animals in both groups were mixed and were fed the same early lactation diet. After approximately five days, the cows were housed in a free stall barn, receiving the early lactation diet until 50 days postpartum.

### Feeding

The nutrient and energy content for the different rations are depicted in Table 1.

**Table 1:** Nutrient and energy content of the dry cow diets in group 1 and group 2 and the lactating diet

Nutrient and energy content	Group 1 “one-phase”	Group 2 “two-phase”		Lactation
		Phase 1^**7**^	Phase 2^**8**^	
**DM (g/kg)**^**1**^	466	458	511	477
**uCP (g/kg DM)**^**2**^	133	118	152	155
**RNB (g/kg DM)**^**3**^	−0.5	−1.7	0.5	0.2
**aNDFom (g/kg DM)**^**4**^	491	534	414	371
**Degradable XS + XZ (g/kg DM)**^**5**^	155	99	190	202
**NEL (MJ/kg DM)**^**6**^	5.95	5.55	6.60	6.95

^1^*DM* Dry matter; ^2^*uCP* Utilisable crude protein; ^3^*RNB* Ruminal nitrogen balance; ^4^aNDFom = Neutral detergent fibre after treatment with amylase and cinefaction;

^5^degradable XS + XZ = degradable starch + sugar; ^6^*NEL* Net energy for lactation, Phase 1^7^ = Date of drying off to two weeks ante partum, Phase 2^8^ = Two weeks ante partum until partus

*Group 1 was fed a one-phase diet* consisting of grass silage (29.4% of dry matter (DM)), corn silage (34.9% of DM), straw (21.2% of DM), post-extraction rapeseed meal (RES) (13.8% of DM) and minerals (0.8% of DM).

*Group 2 was fed a two-phase diet*. For phase 1 (drying off to two weeks ante partum), cows received a diet according to the guidelines of the GfE [37] consisting of grass silage (63.3% of DM), corn silage (12.0% of DM), straw (18.7% of DM) and minerals (1.1% of DM). Two weeks before the calculated calving date, cows received the transition diet consisting of grass silage (27.8% of DM), corn silage (34.5% of DM), straw (6.3% of DM), RES (18.8% of DM), concentrate (11.9% of DM) and minerals (0.8% of DM). The diet was offered ad libitum and fed as a Total Mixed Ration (TMR).

After calving, all cows received the early lactation TMR consisting of grass silage (18.7% of DM), corn silage (42.6% of DM), alfalfa hay (3.2% of DM), RES (16.7% of DM), concentrate (16.2% of DM) and minerals (2.7% of DM).

All diets were mixed once daily and offered in individual feeding troughs equipped with scales. Cows were recognised either by responders (Westfalia Separator Group GmbH, Oelde, Germany/Calan) or chip cards (Waagen Döhrn GmbH & Co. KG, Wesel, Germany). Every day, the DM of the different diets was determined by drying feed samples for 24 h at 105 °C in a drying cabinet. The corrected DM was calculated using the formula in accordance with Weißbach and Kuhla [43]: DM corr = 2.08 + 0.957*DM (%). From the daily values of DM intake, a weekly average was calculated and the energy intake was calculated using the dry mater intake and the estimated values for metabolisable energy. Analysis of the nutrient content was performed according to the guidelines of the Association of German Agricultural Analytic and Research Institutes [38], and method numbers are given. Crude protein and neutral detergent fibre after treatment with amylase and cinefaction (aNDFom) were analysed using methods 4.1.1 and 6.5.1., respectively. Starch was determined using method 7.2.1 and sugar using method 7.1.3. Energy content and utilisable crude protein at the duodenum (uCP) were calculated according to GfE [37].

On an individual level, milk production was recorded for every milking. Fat, protein, and lactose contents in milk were measured weekly. Energy-corrected milk (ECM)/kg per day and cow was calculated as follows: ECM = 0.327 × milk yield (kg/d) + 12.95 × fat (kg/d) + 7.2 × protein (kg/d).

### Blood sampling

From the beginning of drying off onwards, blood samples were taken at weekly intervals, at the day of calving and then at weekly intervals until 6 weeks post partum. Blood samples were collected from the Vena caudalis mediana in EDTA-tubes and centrifuged for 15 min at 9000 rpm. The plasma was aliquoted in Eppendorf vials (1.5 mL, Sarstedt AG & Co. KG, Nürmbrecht, Germany) and stored at − 20 °C until further analysis.

#### Protein content

The protein content of the plasma samples was measured by the biuret method using commercial assay kits (PZ Cormay S.A., Łomianki, Poland).

#### Content of bityrosine bridges

Bityrosine bridges were determined by a spectroflurimetric method in accordance with Rice-Evans et al. [39]. The unaltered plasma sample was excited by light at 325 nm and emission was measured at a wavelength of 410 mm. The spectrofluorimeter (Jasco Corporation, Tokyo, Japan) was standardised to 100 deflections with chinine sulphate (0.1 μg/mL in 0.1 mol/ H_2_SO_4_) at excitation (350 nm) and emission wavelength (445 nm). The results were expressed as mg/g plasmaprotein. The coefficients of variation were: intra-assay 5.9% (*n* = 10) and inter-assay 6.0% (*n* = 10), respectively.

#### The content of formylokinurenine

Formylokinurenine was determined by a spectroflurimetric method in accordance with Rice-Evans et al. [39]. The unaltered plasma sample was excited by light at 360 nm and emission was measured at 454 nm wavelength. The spectrofluorimeter (Jasco Corporation) was standardised as described above [39]. The results were expressed as mg/g plasmaprotein. The coefficients of variation were: intra-assay 6.3% (*n* = 10) and inter-assay 6.5% (*n* = 10).

#### The content of sulfhydryl groups

The concentration of sulfhydryl residues in plasma was measured by spectrophotometry as described in detail by Rice-Evans et al. [39]. A volume of 300 μL 10% (w/v) sodium dodecyl sulphate (SDS, Sigma, Poznań, Poland) in 10 mmol/L sodium phosphate buffer (pH 8.0) was added to 300 μL of sample and mixed precisely. A 2.4 mL of 10 mmol/L sodium phosphate buffer (pH 8.0) was added. Then 300 μL of solution consisting of 20 mg of 5,5-dithiobis-2-nitro benzoate (DTNB, Sigma Aldrich sp. z o.o., Poznań, Poland) in 50 mL of buffer (DTNB) was added and the absorbance was measured at 412 nm. The control sample contained 300 μL of the same buffer instead of DTNB. All samples were incubated for 1 h at 37 °C. After incubation, the absorbance was measured again at 412 nm. The difference in absorbance before and after incubation (after subtracting the respective absorbance of the control) referred to the content of the SH groups. The content was calculated using a standard curve prepared with different dilutions of glutathione (GSH, Sigma Aldrich sp. z o.o.) ranging from 0 to 1 mmol/L in 10 mmol/L sodium phosphate buffer (pH 8.0) and expressed in mmol/g plasmaprotein. The intra-assay coefficient of variation was 6.9% (*n* = 10) and the inter-assay coefficient of variation was 7.1% (*n* = 10).

#### Total antioxidant capacity

Total antioxidant capacity was measured in accordance with the method of Benzie and Strain [40], based on the ferric reducing ability of plasma with some modifications. The changes in absorbance were directly related to the “total” reducing capacity of the electron donating antioxidants present in the examined plasma samples. The working reagent contained 300 mmol/L acetate buffer (pH 3.6), 10 mmol/L 2,4,6-tri-pyridyl-striazine (TPTZ, Sigma Aldrich sp. z o.o.) solution in 40 mmol/L hydrogen chloride and 20 mmol/L FeCl_3_ x 6H_2_O solution in dH_2_O mixed at a ratio of 10:1:1. The working reagent was prepared immediately before use. The working reagent (2250 μL) was mixed with 25 μL of plasma and absorbance was measured at 593 nm. The absorbance of the working reagent alone served as control. After a 10-min incubation-period at room temperature, the absorbance was measured again. The difference in absorbance at zero and at 10 min was compared with a standard curve prepared with ten different dilutions of Fe (II) between 0 and 1000 μmol/L. The results were expressed as μmol/g plasmaprotein. Intra-assay 8.8% (*n* = 10) and inter-assay 8.5% (*n* = 10) coefficients of variation were established.

### Statistical analysis

Statistical analysis was performed applying standard statistical procedures [41] and using the computer program SAS version 9.1 (SAS Institute Corp., Cary, NC USA). All results are presented as least square mean (LSM) ± standard error (SE). For comparing mean concentrations of antioxidant and oxidant blood constituents, a linear mixed effect model for repeated measures was performed (PROC MIXED and method REML) with the following fixed effects: group (one-phase/two-phase) and time periods (week − 5, − 4, − 3, − 2, − 1, Partus, + 1, + 2, + 3, + 4 + 5, + 6; repeated) and group x time interaction. Cows were included as random effect. Analyses were followed by Tukey-Kramer test (LSMEANS/ADJUST = TUCKEY) for adjusted multiple comparisons between the groups and days, respectively. The F- and *p* values for the effects time, group and group × time are presented in the Fig. 1 A-F. Significant differences between the groups (one-phase/two-phase) are indicated by asterix. For the study of temporal patterns, we initially compared all points in time with each other. Due to the correspondingly high number of comparisons and under consideration of relevant time periods, the presentation of the data was limited to the observation of the antepartum and postpartum periods. Accordingly, 5 weeks a.p. was compared with the following time points until partus. To consider the postpartum period, a comparison was made between day of parturition and all following time points. The level of significance was set at *P* < 0.05.

## Supplementary information

**Additional file 1 **Least squares means ± SE of dry matter intake (kg/animal/day), energy intake (MJ/animal/day) and arithmetic mean concentration of bityrosine bridges, formylkinurine, sulfhydryl residues, total antioxidant capacity in blood plasma of “one-phase” (*n* = 20) and “two-phase”(*n* = 20) fed cows from five weeks a.p. up to six weeks p.p.. *P*-value for the time comparison within the groups.

## Data Availability

The datasets used and/or analysed during the current study are available from the corresponding author on reasonable request.

## Abbreviations

aNDFom: Neutral detergent fibre after treatment with amylase and cinefaction
a.p.: Antepartum
BIT: Bityrosine bridges
XS: Starch
XZ: Sugar
DM: Dry matter
DMI: Dry matter intake
DTNB: 5,5-Dithiobis-2-nitro benzoate
ECM: Energy–corrected milk yield
FK: Formylokinurenine
GPx: Glutathione peroxidase
GSH: Glutathione
LSM: Least square mean
NDF: Neutral detergent fibre
NEL: Net energy for lactation
p.p.: Postpartum
RES: Post-extraction rapeseed meal
RNB: Ruminal nitrogen balance
ROS: Reactive oxygen species
SDS: Sodium dodecyl sulphate
SE: Standard error
SH: sulfhydryl residues
TAC: Total antioxidant capacity
TMR: Total mixed ration
TPTZ: 2,4,6-Tri-pyridyl-striazine
uCP: Utilisable crude protein
