# Supplementary material for: Influence of two different feeding strategies in the dry period on dry matter intake and plasma protein peroxidative and antioxidative profile during dry period and early lactation
Source: BMC Vet Res. 2020 May 13;16:134. doi: 10.1186/s12917-020-02347-x (PMC7222456; doi:10.1186/s12917-020-02347-x)
Supplement: Supplementary file 1 — Additional file 1 Least squares means ± SE of dry matter intake (kg/animal/day), energy intake (MJ/animal/day) and arithmetic mean concentration of bityrosine bridges, formylkinurine, sulfhydryl residues, total antioxidant capacity in blood plasma of “one-phase” (n = 20) and “two-phase”(n = 20) fed cows from five weeks a.p. up to six weeks p.p.. P-value for the time comparison within the groups. [file 12917_2020_2347_MOESM1_ESM.docx]

Additional file 1:

Table: Least squares means (LSM) ± SE of dry matter intake (kg/animal/day), energy intake (MJ/animal/day) and concentration of bityrosine bridges, formylkinurine, sulfhydryl residues, total antioxidant capacity in blood plasma of “one-phase” (n = 20) and “two-phase”(n = 20) fed cows from five weeks a.p. up to six weeks p.p.. *P*-value for the time comparison within the groups.

|  | | | Weeks related to partus | | | | | | | | | | | | | | | | | | | | | | |
| --- | --- | --- | --- | --- | --- | --- | --- | --- | --- | --- | --- | --- | --- | --- | --- | --- | --- | --- | --- | --- | --- | --- | --- | --- | --- |
|  |  |  |  | |  | |  | |  | |  | |  | |  | |  | |  | |  | |  | |  |
| **Parameter** | Group |  | -5 | | -4 | | -3 | | -2 | | -1 | | Partus | | 1 | | 2 | | 3 | | 4 | | 5 | | 6 |
| **Dry matter**  **Intake**  (kg/animal/day) | one-phase | LSM | 12.53 | | 12.89 | | 12.42 | | 12.13 | | 11.75 | | 10.10  ^.a^ | | 11.33 | | 13.60 | | 15.96 | | 18.16 | | 18.34 | | 18.99 |
|  |  | SE | 0.70 | | 0.70 | | 0.70 | | 0.70 | | 0.70 | | 0.71 | | 0.72 | | 0.71 | | 0.70 | | 0.70 | | 0.70 | | 0.70 |
|  | *P*-value* | |  | | 0.6138 | | 0.8770 | | 0.5764 | | 0.2749 | | 0.0010 | | 0.1009 | | <0.0001 | | <0.0001 | | <0.0001 | | <0.0001 | | <0.0001 |
|  | two-phase | LSM | 10.82 | | 10.70 | | 10.47 | | 11.68 | | 13.07 | | 11.52 | | 11.34 | | 14.57 | | 17.27 | | 18.66 | | 19.10 | | 20.43 |
|  |  | SE | 0.66 | | 0.66 | | 0.66 | | 0.66 | | 0.66 | | 0.66 | | 0.66 | | 0.66 | | 0.66 | | 0.66 | | 0.67 | | 0.66 |
|  | *P*-value* | |  | | 0.8564 | | 0.5921 | | 0.1890 | | 0.0007 | | 0.2845 | | 0.7822 | | <0.0001 | | <0.0001 | | <0.0001 | | <0.0001 | | <0.0001 |
|  |  |  |  | |  | |  | |  | |  | |  | |  | |  | |  | |  | |  | |  |
| **Energy**  **Intake**  (MJ/animal/day) | one-phase | LSM | 74.26 | | 76.26 | | 73.98 | | 72.30 | | 69.92 | | 61.03 | | 78.70 | | 94.54 | | 110.96 | | 126.49 | | 127.85 | | 132.37 |
|  |  | SE | 4.74 | | 4.74 | | 4.74 | | 4.74 | | 4.74 | | 4.81 | | 4.89 | | 4.81 | | 4.74 | | 4.74 | | 4.74 | | 4.74 |
|  | *P*-value* | |  | | 0.6884 | | 0.9566 | | 0.6966 | | 0.3872 | | 0.0097 | | 0.0008 | | <0.0001 | | <0.0001 | | <0.0001 | | <0.0001 | | <0.0001 |
|  | two-phase | LSM | 59.12 | | 58.48 | | 57.81 | | 69.23 | | 85.77 | | 76.60 | | 78.96 | | 102.43 | | 120.20 | | 130.13 | | 133.11 | | 142.62 |
|  |  | SE | 4.54 | | 4.54 | | 4.54 | | 4.54 | | 4.54 | | 4.54 | | 4.54 | | 4.60 | | 4.54 | | 4.54 | | 4.60 | | 4.54 |
|  | *P*-value* | |  | | 0.8893 | | 0.7764 | | 0.0289 | | <0.0001 | | 0.0002 | | 0.6081 | | <0.0001 | | <0.0001 | | <0.0001 | | <0.0001 | | <0.0001 |
|  |  |  |  | |  | |  | |  | |  | |  | |  | |  | |  | |  | |  | |  |
| **Bityrosine bridges**  (mg x10^-3^/g Plasmaprotein) | one-phase | LSM | 2.86 | | 2.83 | | 2.85 | | 2.71 | | 2.54 | | 1.99 | | 2.70 | | 2.59 | | 3.07 | | 3.43 | | 3.40 | | 3.47 |
|  |  | SE | 0.15 | | 0.15 | | 0.15 | | 0.15 | | 0.15 | | 0.15 | | 0.15 | | 0.15 | | 0.15 | | 0.15 | | 0.15 | | 0.15 |
|  | *P*-value* | |  | | 0.8816 | | 0.9764 | | 0.4154 | | 0.0744 | | <0.0001 | | <0.0001 | | 0.0008 | | <0.0001 | | <0.0001 | | <0.0001 | | <0.0001 |
|  | two-phase | LSM | 3.12 | | 3.25 | | 3.50 | | 3.40 | | 3.17 | | 2.55 | | 2.95 | | 3.07 | | 3.25 | | 3.63 | | 3.88 | | 3.77 |
|  |  | SE | 0.19 | | 0.19 | | 0.19 | | 0.19 | | 0.19 | | 0.19 | | 0.19 | | 0.19 | | 0.19 | | 0.19 | | 0.19 | | 0.19 |
|  | *P*-value* | |  | | 0.5214 | | 0.0606 | | 0.1662 | | 0.8206 | | 0.0046 | | 0.0434 | | 0.0101 | | 0.0005 | | <0.0001 | | <0.0001 | | <0.0001 |
|  |  |  |  | |  | |  | |  | |  | |  | |  | |  | |  | |  | |  | |  |
| **Formyl-**  **Kinurine**  (mg x10^-3^/g  Plasmaprotein) | one-phase | LSM | 91.70 | | 92.80 | | 94.30 | | 92.00 | | 86.80 | | 68.00 | | 86.80 | | 84.90 | | 102.60 | | 111.80 | | 110.50 | | 110.50 |
|  |  | SE | 5.32 | | 5.32 | | 5.32 | | 5.32 | | 5.32 | | 5.32 | | 5.32 | | 5.32 | | 5.32 | | 5.32 | | 5.32 | | 5.32 |
|  | *P*-value* | |  | | 0.8521 | | 0.6593 | | 0.9574 | | 0.4020 | | <0.0001 | | 0.0016 | | 0.0046 | | <0.0001 | | <0.0001 | | <0.0001 | | <0.0001 |
|  | two-phase | LSM | 89.10 | | 86.40 | | 102.00 | | 101.50 | | 100.60 | | 80.20 | | 84.90 | | 98.20 | | 107.30 | | 113.70 | | 121.70 | | 116.00 |
|  |  | SE | 7.77 | | 7.77 | | 7.77 | | 7.77 | | 7.77 | | 7.77 | | 7.77 | | 7.77 | | 7.77 | | 7.77 | | 7.77 | | 7.77 |
|  | *P*-value* | |  | | 0.7672 | | 0.1766 | | 0.1940 | | 0.2294 | | 0.3488 | | 0.3642 | | 0.0596 | | 0.0047 | | 0.0005 | | <0.0001 | | 0.0002 |
|  |  |  |  | |  | |  | |  | |  | |  | |  | |  | |  | |  | |  | |  |
| **Sulfhydryl**  **groups**  (mmol x10^-3^/g Plasmaprotein) | one-phase | LSM | 6.60 | | 6.40 | | 7.00 | | 6.40 | | 7.00 | | 7.20 | | 6.70 | | 6.00 | | 6.30 | | 6.50 | | 6.70 | | 6.40 |
|  |  | SE | 0.56 | | 0.56 | | 0.56 | | 0.56 | | 0.56 | | 0.56 | | 0.56 | | 0.56 | | 0.56 | | 0.56 | | 0.56 | | 0.56 |
|  | *P*-value* | |  | | 0.6228 | | 0.1529 | | 0.7247 | | 0.1987 | | 0.0294 | | 0.0889 | | <0.001 | | 0.0016 | | 0.0166 | | 0.0637 | | 0.006 |
|  | two-phase | LSM | 6.00 | | 6.20 | | 6.60 | | 6.80 | | 6.50 | | 6.90 | | 6.40 | | 6.30 | | 6.30 | | 6.60 | | 6.50 | | 6.50 |
|  |  | SE | 0.43 | | 0.43 | | 0.43 | | 0.43 | | 0.43 | | 0.43 | | 0.43 | | 0.43 | | 0.43 | | 0.43 | | 0.43 | | 0.43 |
|  | *P*-value* | |  | | 0.4776 | | 0.0306 | | 0.0055 | | 0.1097 | | 0.0010 | | 0.0767 | | 0.0173 | | 0.0354 | | 0.2231 | | 0.0981 | | 0.0988 |
|  |  |  |  | |  | |  | |  | |  | |  | |  | |  | |  | |  | |  | |  |
| **Total antioxidant capacity**  (µmol/g Plasmaprotein) | one-phase | LSM | 4.40 | | 4.34 | | 4.77 | | 4.33 | | 4.64 | | 4.89 | | 4.50 | | 4.48 | | 4.61 | | 4.70 | | 4.53 | | 4.54 |
|  |  | SE | 0.28 | | 0.28 | | 0.28 | | 0.28 | | 0.28 | | 0.28 | | 0.28 | | 0.28 | | 0.28 | | 0.28 | | 0.28 | | 0.28 |
|  | *P*-value* | |  | | 0.8385 | | 0.1495 | | 0.7929 | | 0.3374 | | 0.0561 | | 0.1328 | | 0.1099 | | 0.2824 | | 0.4673 | | 0.1672 | | 0.1739 |
|  | two-phase | LSM | 4.35 | | 4.40 | | 4.69 | | 4.99 | | 4.84 | | 5.03 | | 4.42 | | 4.33 | | 4.44 | | 4.31 | | 4.62 | | 4.56 |
|  |  | SE | 0.37 | 0.37 | | 0.37 | | 0.37 | | 0.37 | | 0.37 | | 0.37 | | 0.37 | | 0.37 | | 0.37 | | 0.37 | | 0.37 | |
|  | *P*-value* | |  | 0.8290 | | 0.1720 | | 0.0104 | | 0.0492 | | 0.0062 | | 0.0149 | | 0.0049 | | 0.0172 | | 0.0041 | | 0.0949 | | 0.0577 | |
|  |  |  |  |  | |  | |  | |  | |  | |  | |  | |  | |  | |  | |  | |

**P*-value for the time comparison within the groups in the following order -5 to -4; -5 to -3; -5 to -2; -5 to -1; -5 to partus; partus to 1; partus to 2; partus to 3; partus to 4; partus to 5; partus to 6
